# Mitomycin and 5‐fluorouracil for second‐line treatment of metastatic squamous cell carcinomas of the anal canal

**DOI:** 10.1002/cam4.2558

**Published:** 2019-09-16

**Authors:** Angélique Saint, Ludovic Evesque, Alexander T. Falk, Gérard Cavaglione, Lucile Montagne, Karen Benezery, Eric Francois

**Affiliations:** ^1^ Digestive Oncology Department of Medical Oncology Centre Antoine Lacassagne Nice France; ^2^ Department of Radiation Oncology Centre Antoine‐Lacassagne Nice France

**Keywords:** 5‐fluorouracil, anal canal, cancer, chemotherapy, metastasis, mitomycin

## Abstract

**Background:**

Metastatic squamous cell carcinomas (SCC) of the anal canal are rare and there is no international consensus on their second‐line management. 5‐Fluorouracil (5‐FU) and mitomycin in combination with radiotherapy is the standard for locally advanced forms but its efficacy in metastatic stage has never been evaluated.

**Patients and methods:**

We report a retrospective analysis of patients treated with 5‐FU and mitomycin from 2000 to 2017 in our institution for a metastatic SCC of the anal canal after failure of platinum‐based regimen. The main outcome was progression‐free survival (PFS) and the secondary outcomes were overall survival (OS), response rate, and toxicity.

**Results:**

Nineteen patients, 15 women and four men, with a median age of 57 years were identified (range, 40‐79 years). Patients received a median of three cycles (1‐7) of mitomycin 5‐FU. A dose reduction was necessary in six patients (31.6%), one patient had to discontinue treatment following toxicity and no death was due to treatment toxicity was reported. An objective response was observed in five patients (26.4%, 95% CI 6.6‐46.2) including one complete response, six patients (31.6%, 95% CI 10.7‐52.5) showed tumor stabilization. Median PFS and OS were 3 months [95% CI 1‐5] and 7 months [95% CI 2.2‐11.8]. Responder had a median duration of response of 4 months [95% CI 1.8‐6.1] and one patient had 23 months duration of response. No significant difference was noted for PFS and OS for patients previously treated with mitomycin and 5‐FU at a local stage.

**Conclusion:**

Mitomycin and 5‐FU regimen provides tumor control with acceptable tolerance. It is an option for patients with metastatic SCC of the anal canal after failure of platinum‐based chemotherapy. [Correction added on 9 October 2019, after first online publication: '5‐FU' was inadvertently removed from the Results and Conclusion and has now been added to the text.]

## Graphical abstract

5‐Fluorouracil and mitomycin in combination with radiotherapy is the standard for locally advanced squamous cell carcinomas of the anal canal and we have evaluated its efficacy in metastatic stage. This study is a retrospective analysis of patients treated with and mitomycin from 2000 to 2017 in our institution for a metastatic SCC of the anal canal after failure of platinum‐based regimen. Mitomycin and 5‐FU provides a Response Evaluation Criteria in Solid Tumors response in nearly a quarter of patients with acceptable toxicity. It is a reasonable therapeutic option after failure of a first‐line chemotherapy in the absence of other therapeutic option.

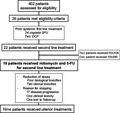

## 1 INTRODUCTION

Squamous cell carcinoma of the anal canal (SCCA) is a rare disease with an estimated worldwide annual incidence of around 27 000 cases.^1^ Metastatic disease is less frequent and representing approximately 20% of patients, whether diagnosed asynchronous or metachronous metastases after curative treatment of a primary tumor.^2^, ^3^, ^4^

Due to the low frequency of metastatic forms, the level of proof of the current treatment recommendations is low. The chemotherapy regimen associating cisplatin and 5‐fluorouracil (5‐FU) has so far constituted the standard first‐line treatment although based on retrospective data involving a small number of patients.^5^, ^6^ The data in the literature reveal high response rates (30%‐75%) but short response durations (5.8‐8 months).^7^ For very selected patients with oligometastasic disease, multimodal management could enhance tumor control and patient survival.^8^ Recently, the results of the phase 2 multicentric clinical trial Epitopes‐HPV02 define tri‐chemotherapy using docetaxel, cisplatin, and 5‐FU (DCF) as a new option in first‐line treatment for patients with an Eastern Cooperative Oncology Group of 0‐1 with results showing an 86% objective response rate, 11 months progression‐free survival (PFS), and an overall survival rate (OS) at 12 months of 83%.^9^ In addition, the presentation of the results of the randomized phase 2 trial interAACT at the last European Society for Medical Oncology (ESMO) congress demonstrated similar response rates to treatment between cisplatin plus 5‐FU compared to carboplatin plus paclitaxel regimen (57.1% vs 59.0%) but less toxicities with carboplatin plus paclitaxel regimen and established another potential standard of care for the first‐line treatment of inoperable locally advanced or metastatic SCCA.^10^ Regarding the second‐line treatment, no international consensus has so far emerged. In 2018, National Comprehensive Cancer Network Clinical Practice Guidelines added two programmed cell death protein‐1 (PD‐1) inhibitors, as option based on the encouraging results of a multicenter phase 2 and a phase 1b trials.^11^, ^12^, ^13^

[Correction added on 9 October 2019, after first online publication: In the preceding sentence, "nivolumab and pembrolizumab, two programmed cell death protein‐1 (PD‐1) and programmed cell death ligand‐1 Programmed death‐ligand (PDL‐1) inhibitors, as options" has been changed to "two programmed cell death protein‐1 (PD‐1) inhibitors, as option"] However, pending more robust results, checkpoint inhibitors are not yet recommended in Europe for this indication.^14^

Numerous studies have assessed the association of 5‐FU and mitomycin‐C in patients presenting heavily pretreated metastatic colorectal cancer. In view of the efficacy and the acceptable—mainly hematologic—toxicity, this combination has been suggested as an alternative therapeutic option for this indication.^15^ Fluoropyrimidines in association with mitomycin act synergistically in vitro.^16^, ^17^ 5‐Fluorouracil mitomycin regimen, in association with radiotherapy, constitutes the gold standard for locally advanced forms.^18^, ^19^ However, to our knowledge, its advantages in a metastatic situation have never been evaluated.

We report here a retrospective analysis of patients treated by 5‐FU and mitomycin between 2000 and 2017 at our institution in the second‐line treatment for metastatic SCCA after failure of cisplatin 5‐FU regimen.

## 2 MATERIALS AND METHODS

### 2.1 Patients

We retrospectively reviewed all the medical files extracted from our institutional database and found 402 patients with anal canal cancer treated between January 2000 and January 2017.

Inclusion criteria were as follows: Histological confirmed SCCA, inoperable locally recurrent or metastatic disease, measurable disease by Response Evaluation Criteria in Solid Tumors (RECIST) criteria, and first‐line metastatic treatment combining cisplatin and 5‐FU. Patients refusing the use their personal data to be used for scientific research, patients with a previous history of another cancer less than 5 years with the exception of basal cell or squamous cell skin carcinomas, and patients lost to follow‐up were excluded from the analysis.

The medical files of each patient were reviewed. The starting date of treatment and date of progression or, when relevant, the date of the last delivery of the treatment regimen were collected. The dates of the most recent information and health status of the patient at that date were also analyzed, as well the clinical characteristics and biological data at initiation of treatment and at each cycle. Treatment tolerance and chemotherapy dose adjustments data were gathered.

### 2.2 Chemotherapy

The chemotherapy regimen comprised administration of mitomycin 10 mg/m^2^ for 15 minutes followed by continuous infusion of 5‐FU 1000 mg/m^2^/d for 96 hours, repeated every 28 days. Chemotherapy was continued until progression, toxicity, or patient refusal to continue treatment. Chemotherapy initiation was discussed beforehand at a multidisciplinary meeting. During the treatment period, patients underwent morphologic assessment by thoraco‐abdomino‐pelvic computerized tomography scan with intravenous injection of iodine‐containing contrast medium every 2 months throughout the duration of treatment.

### 2.3 Statistical analysis

Primary outcome was PFS. Secondary outcomes include OS, response rate, and toxicity. Response rate was defined according to RECIST 1.1 criteria.^20^ Toxicity was graded before each cycle using the National Cancer Institute (CTCAE version 4.0) criteria.

Descriptions of the population and the different parameters studied were presented using absolute and relative frequencies for qualitative data and were summarized using descriptive statistics such as median and extreme for qualitative data. Progression‐free survival was defined as the length of time between the date of initiation of second‐line chemotherapy and the date of progression. Overall survival was defined as the length of time between initiation of second‐line chemotherapy and the date of the latest patient information. Patients presenting no progression and patients still alive at the date of the latest information during the last follow‐up were censored at that date. Survival data with 95% CI were calculated and reproduced graphically at different times using the Kaplan‐Meier method. Survival graphs were compared using the log‐rank test. Analysis was performed using SPPS software, version 22.0.

This study has been declared to the French data protection authority (CNIL), reference number 18006.

## 3 RESULTS

**Figure 1:**
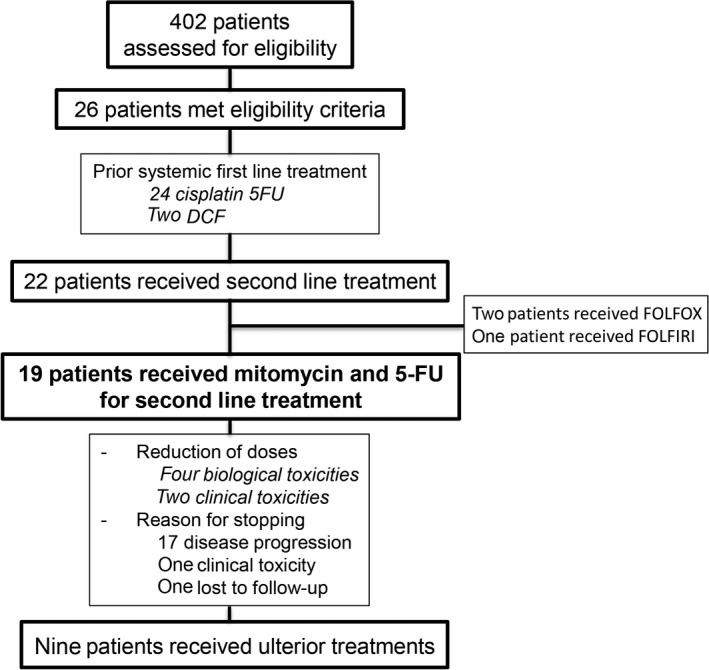
Flowchart

A total of 26 patients matching inclusion criteria were identified. After failure of cisplatin‐5‐FU, 22 patients received a second line regimen: 19 patients received mitomycin and 5‐FU, two patients received FOLFOX, and one patient FOLFIRI (Figure1). Among the 19 patients who received mitomycin and 5‐FU, we identified 15 women (79%) and four men (21%), with a median age 57 years (range: 40‐79). Two patients (10.5%) presented immunosuppression. Fourteen (74%) and five (26%) patients had metachronous and synchronous metastasis, respectively. All patients with metachronous metastases previously had received concomitant radio‐chemotherapy with 5‐FU mitomycin for local disease treatment.

For the first‐line metastatic chemotherapy, 17 patients (89.5%) received cisplatin‐5 FU combination and two patients (10.5%) received tri‐chemotherapy with DCF. First‐line treatment was discontinued due to progression for 10 patients (52.6%), clinical toxicities for five patients (26.4%), biological toxicities for two patients (10.5%), and at their request for two patients (10.5%). Median PFS for first‐line chemotherapy was 5 months [95% CI 2.1‐7.8].

Patient characteristics at the time of second‐line treatment are shown in Table 1. Among these patients, 13 (68.5%) had a performance status ≤1 at treatment initiation. Multivisceral metastatic disease was observed in 11 patients (57.9%) with more than two‐thirds of whom had liver metastasis.

**Table 1:** Clinical characteristics of the patients at beginning of second‐line

	n = 19 (%)
Median age, y (range)	57 (40‐79)
Sex	
Male	4 (21)
Female	15 (79)
Immunodepression	2 (10.5)
HIV	1 (5.2)
Kidney graft	1 (5.3)
ECOG, performance status	
0	1 (5.3)
1	12 (63.2)
2	4 (21)
Unknown	2 (10.5)
Tumor status at diagnosis	
Locally advanced	14 (74)
Synchronous metastasis	5 (26)
Distribution of unresectable disease	
Local recurrence + distant metastasis	5 (26)
Distant metastasis	19 (100)
Number of metastatic sites	
1	8 (42.1)
2	8 (42.1)
≥3	3 (15.8)
Sites of distant metastasis	
Lymph node	8 (42.1)
Liver	12 (63.2)
Lung	8 (42.1)
Peritoneum	3 (15.8)
Bone	2 (10.5)
Other	1 (5.3)
Prior radiation	18 (94.7)
Systemic treatment at locally‐advanced stage	
5‐FU + mitomycin	7 (37)
5‐FU + platinum	7 (37)
First systemic treatment at unresectable stage	
5‐FU + platinum	17 (89.5)
5‐FU + docetaxel + platinum	2 (10.5)

Abbreviation: ECOG, Eastern Cooperative Oncology Group; SFU, 5‐fluorouracil.

Patients received a median of three cycles.^1^, ^2^, ^3^, ^4^, ^5^, ^6^, ^7^ The most frequently encountered nonhematological toxicities were mainly grade I‐II digestive toxicities such as diarrhea and/or mucositis in 63.2% of patients. Six patients (31.6%) presented grade I‐II hematological toxicities and three patients (16%) presented grade III‐IV with febrile neutropenia. A dose reduction was necessary in six patients (31.6%). One patient (5.3%) had to discontinue treatment following toxicity and no death was due to treatment toxicity.

An objective response was observed in five patients (26.4%, 95% CI 6.6‐46.2). Of these, four (21.1%) were partial responses and one (5.3%) a complete response. At first assessment, six patients showed tumor stabilization (31.6%, 95% CI 10.7‐52.5) (Table 2).

**Table 2:** Antitumor activity

Variable	n = 19
Confirmed objective response rate—%	26.4
Confirmed best overall response—no (%)	
Complete response	1 (5.3)
Partial response	4 (21,1)
Stable disease	6 (31.6)
Progressive disease	8 (42)

**Figure 2:**
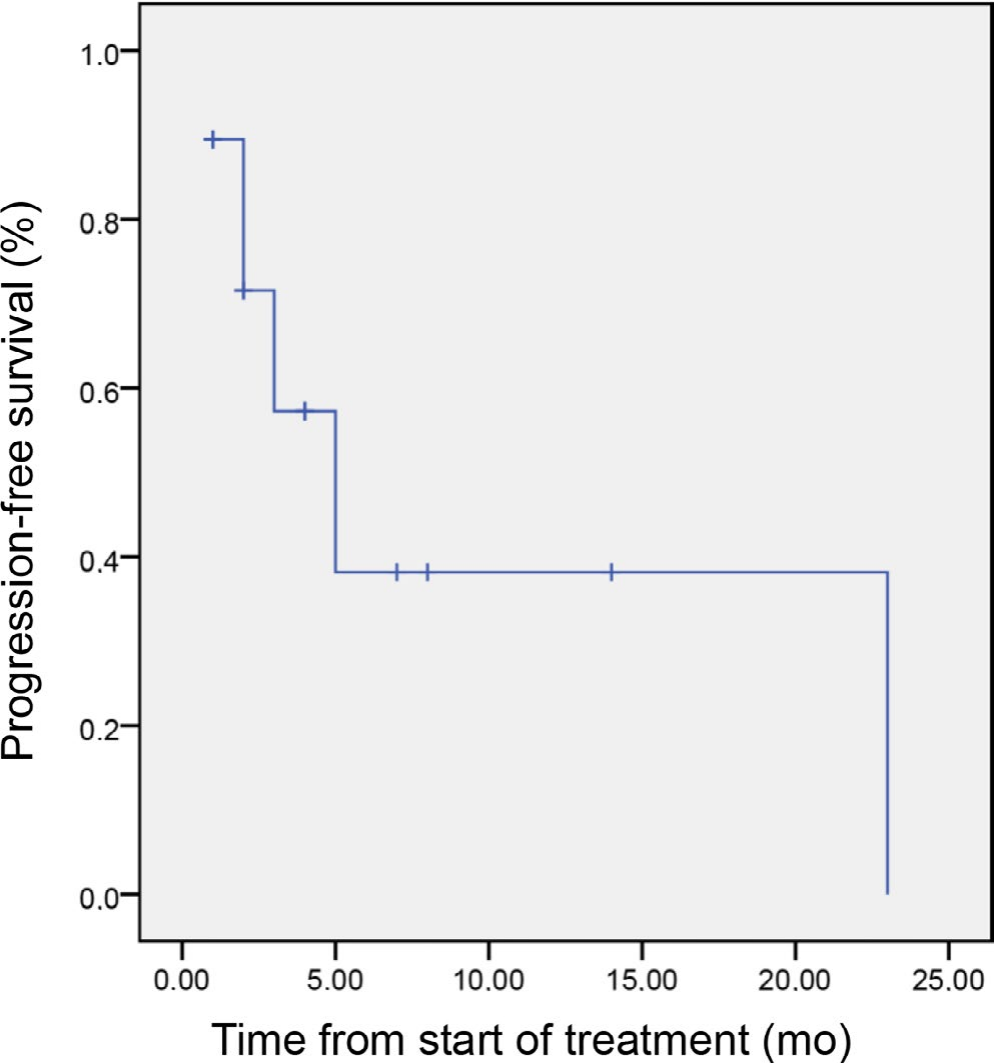
Progression‐free survival

**Figure 3:**
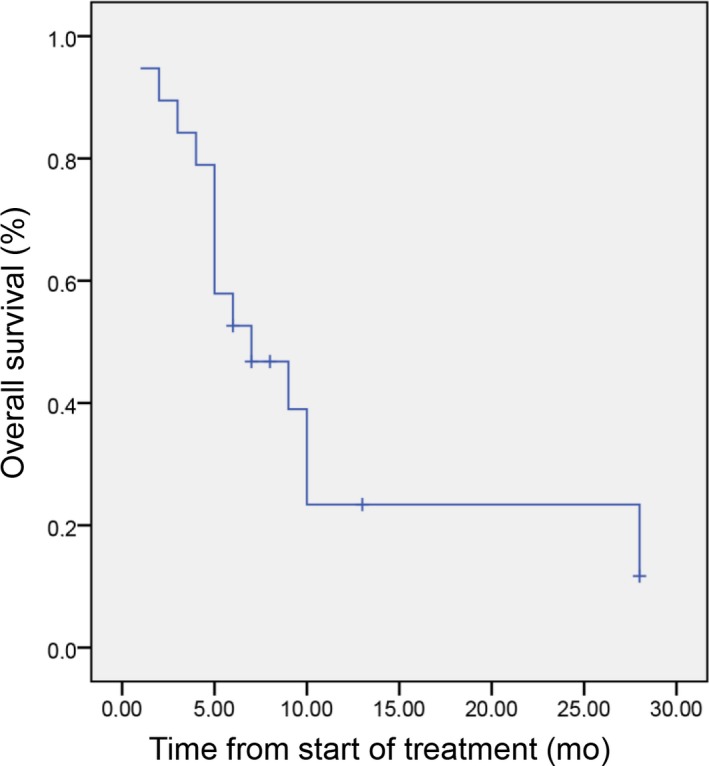
Overall survival

Median PFS was 3 months [95% CI 1‐5] (Figure 2) and OS was 7 months [95% CI 2.2‐11.8] (Figure 3). For patients previously treated at a local stage with mitomycin and 5‐FU during their concomitant radio‐chemotherapy, no significant difference was noted for PFS (4 months [95% CI 2‐6; *P* = .6]) and OS (10 months [95% CI 7.5‐20; *P* = .7]) compared to patients with synchronous metastatic disease. Median duration of response for responding patients was 4 months [95% CI 1.8‐6.1]. One patient had a 23 months response duration.

A third‐line chemotherapy was initiated for 45% of patients. 15% of patients have received vinorelbine, 10% a FOLFIRI regimen, 5% capecitabine, 5% a FOLFOX regimen, 5% paclitaxel and 5% the association carboplatin plus paclitaxel. One patient (5%) received a fourth line of treatment (Table 3).

**Table 3:** Later systemic treatments. FOLFIRI (irinotecan + folinic acid + 5‐fluorouracil), FOLFOX (oxaliplatin + folinic acid + 5‐fluorouracil)

Variable	n = 19
Number of different systemic treatments n (%)	
2	9 (45)
3	9 (45)
4	1 (5)
Third systemic treatment	
Vinorelbine	3 (15)
Folfiri	2 (10)
Capecitabine	1 (5)
Folfox	1 (5)
Paclitaxel	1 (5)
Carboplatin paclitaxel	1 (5)
Fourth systemic treatment	
Docetaxel‐5‐FU‐cisplatin	1 (5)

## 4 DISCUSSION

There is very little evidence in the literature regarding the second‐line management of advanced SCCA and prognosis of these patients remains poor. Despite the limitations and biases inherent to a small, retrospective, and single‐institution analysis, this study is the first to assess the potential benefit of chemotherapy combination of mitomycin with 5‐FU as second‐line in patients presenting an inoperable locally advanced or metastatic SCCA.

An objective response was observed in a quarter of our patients. Despite this promising response rate, median PFS was 3 months and OS was estimated at only 7 months. However, all our patients received second‐line therapy following failure of the first‐line treatment by cisplatin and 5‐FU. In metastatic head and neck^21^ or uterine‐cervix SCCs^22^ after failure of platinum regimen, response rates (0‐32%), PFS (1.9‐5.2 months), and OS (3.7‐9.3 months) showed similar ranges. In the specific framework of SCCA, the efficacy of paclitaxel chemotherapy after failure of cisplatin‐5‐FU combination has been reported in a series of seven cases with a partial response observed in four patients.^23^ Another study assessing carboplatin‐paclitaxel combination delivered to six patients as second‐line treatment reported only one stabilization.^24^ More recently, a retrospective analysis of 64 patients with inoperable locally advanced SCCA showed that second‐line systemic treatment was administrated to approximately one‐third of patients after failure of a combination of platinum agent plus 5‐FU. Progression‐free survival after second‐line chemotherapy was also 3.2 months (IQR, 2.5‐7.1 months) in this case.^25^

We may think that the change in future practices in the first‐line treatment using carboplatin plus paclitaxel or DCF regimen could reduce the use of platinum salts and taxanes at progression justifying the use of other molecules. In our study, only two patients received the first‐line chemotherapy with DCF which is a limitation. In Epitopes‐HPV02 study, 89% of patients presented an objective response after only eight cycles of modified DCF^9^ but, to date, there are no data about the efficacy of taxanes reintroduction after failure of a docetaxel or paclitaxel‐based chemotherapy.

Experience using anti‐EGFRs in combination with cytotoxic agents after failure of first‐line chemotherapy has shown tumor response rates of around 30% and a PFS rate of 7 months on small study cohorts, these results are promising but need to be confirmed.^26^, ^27^ Immunotherapy is another interesting approach. A recent phase II study assessed the efficacy of using PD1 inhibitor (nivolumab) after failure of at least one line of chemotherapy in metastatic patients.^12^ The main objective of this study was the tumoral response rate which was 24%, median response duration was 5.8 months, PFS and OS were 4.1 and 11.5 months, respectively. A second phase Ib study reported interesting results in terms of tolerance and antitumor activity in a population of patients with locally advanced or metastatic SCCA overexpressing PDL‐1 and receiving pembrolizumab after failure of at least one line of chemotherapy.^13^ These results are not very far from ours regarding the tumor response rate but appear to indicate an advantage in favor of immunotherapy in terms of PFS and OS. It should be noted that all patients in these prospective trials exhibited a preserved general status at inclusion. In our study, a third of patients had a performans status of 2, which may have had a negative impact on our results. Currently, there is no predictive therapeutic response marker for PD1 or PDL‐1 inhibitors. The potential benefit of immunotherapy needs to be confirmed and additional investigations are required in order to determine which patients might benefit from immunotherapy in this location. We need to extend our understanding of tumor biology to identify predictive response biomarkers likely to assist in treatment selection. One of the potential ways to improve immunotherapy outcomes may be treatment combinations. Recently, a single arm, single center, phase 2 trial studied the potential effectiveness of nivolumab in combination with an HPV‐16 vaccine in 24 patients with incurable HPV‐16‐positive cancer including one anal cancer.^28^ The overall response rate was 33% (90% CI, 19%‐50%) including a durable complete response for the patient with anal canal cancer. median progression free survival and median overall survival were 2.7 and 17.5 months, respectively, in this case. These very encouraging results must be confirmed by a randomized clinical trial.

Some studies are in progress, in particular the CARACAS study evaluating the efficacy of combining cetuximab with avelumab vs avelumab as monotherapy for locally advanced or metastatic anal carcinomas who progressed after at least one line of treatment.^29^

In the present study, we found no significant difference in terms of efficacy in the patient sub‐group receiving mitomycin 5‐FU in combination with radiotherapy during tumor management at a localized stage. Moreover, we have not highlighted an increase in toxicity when this chemotherapy was reintroduced.

Concerning the treatment tolerance, the retrospective nature of the study induced a bias due to the possibly incomplete medical files resulting in underestimation of the true level of toxicity. However, toxicities reported in our study with this therapeutic combination are concordant with the results already reported in the literature. Kang et al, for example, in a population treated for third‐line metastatic colorectal adenocarcinomas, reported mainly grades III‐IV hematologic toxicities represented by neutropenia and thrombopenia in 10.8% and 8.8% of patients, respectively.^30^ Grades III‐IV nonhematological toxicities comprised nausea and vomiting in 4% and mucositis in 2.2% of patients. Two phase II trials which prospectively enrolled patients with colorectal cancer for third line chemotherapy also reported mainly hematological toxicity.^31^, ^32^

## 5 CONCLUSION

In our study, the combination of mitomycin and 5‐FU provides a RECIST response in nearly a quarter of patients despite a relatively short median response time. Toxicity, although not negligible, appears acceptable in patients who remain in good general condition and for whom there is no alternative treatment. In view of these preliminary data, and the absence of a clear established therapeutic alternative, this regimen appears to our team to constitute a reasonable therapeutic option for patients after failure of a first‐line chemotherapy with 5‐FU and cisplatin. This work highlights the lack of robust data in this situation and the need to undertake randomized clinical trials evaluating, in particular, combinations of treatments with checkpoints inhibitors.

## CONFLICT OF INTEREST

The authors and planners have disclosed no potential conflict of interest, financial, or otherwise.

## Data Availability

The data that support the findings of this study are openly available in Pubmed https://www.ncbi.nlm.nih.gov/pubmed/.

## References

[1] De Martel C , Ferlay J , Franceschi S , et al. Global burden of cancers attributable to infections in 2008: a review and synthetic analysis. Lancet Oncol. 2012;13(6):607‐615. 22575588 10.1016/S1470-2045(12)70137-7

[2] Das P , Bhatia S , Eng C , et al. Predictors and patterns of recurrence after definitive chemoradiation for anal cancer. Int J Radiat Oncol Biol Phys;68(3):794‐800. 17379452 10.1016/j.ijrobp.2006.12.052

[3] Eng C . Anal cancer: current and future methodology. Cancer Invest. 2006;24(5):535‐544. 16939964 10.1080/07357900600815208

[4] Gunderson LL , Winter KA , Ajani JA , et al. Long‐term update of US GI intergroup RTOG 98–11 phase III trial for anal carcinoma: survival, relapse, and colostomy failure with concurrent chemoradiation involving fluorouracil/mitomycin versus fluorouracil/cisplatin. J Clin Oncol. 2012;30(35):4344‐4351. 23150707 10.1200/JCO.2012.43.8085 PMC3515768

[5] Ajani JA , Carrasco CH , Jackson DE , Wallace S . Combination of cisplatin plus fluoropyrimidine chemotherapy effective against liver metastases from carcinoma of the anal canal. Am J Med;87(2):221‐224. 2527006 10.1016/s0002-9343(89)80702-8

[6] Faivre C , Rougier P , Ducreux M , et al. 5‐fluorouracile and cisplatinum combination chemotherapy for metastatic squamous‐cell anal cancer. Bull Cancer. 1999;86(10):861‐865. 10572237

[7] Sclafani F , Rao S . Systemic therapies for advanced squamous cell anal cancer. Curr Oncol Rep. 2018;20(7):53. 29728940 10.1007/s11912-018-0698-6

[8] Evesque L , Benezery K , Follana P , et al. Multimodal therapy of squamous cell carcinoma of the anus with distant metastasis: a single‐institution experience. Dis Colon Rectum. 2017;60(8):785‐791. 28682963 10.1097/DCR.0000000000000827

[9] Kim S , François E , André T , et al. Docetaxel, cisplatin, and fluorouracil chemotherapy for metastatic or unresectable locally recurrent anal squamous cell carcinoma (Epitopes‐HPV02): a multicentre, single‐arm, phase 2 study. Lancet Oncol. 2018;19(8):1094‐1106. 30042063 10.1016/S1470-2045(18)30321-8

[10] Carboplatin Plus Paclitaxel Respresents a New Standard of Care for Patients with Squamous Cell Carcinoma of the Anal Canal. ESMO. https://www.esmo.org/Oncology-News/InterAACT-inoperable-locally-recurrent-metastatic-anal-cancer-Rao. Accessed October 24, 2018.

[11] Benson AB , Venook AP , Al‐Hawary MM , et al. Anal carcinoma, version 2.2018, NCCN clinical practice guidelines in oncology. J Natl Compr Canc Netw. 2018;16(7):852‐871. 30006428 10.6004/jnccn.2018.0060 PMC10181270

[12] Morris VK , Salem ME , Nimeiri H , et al. Nivolumab for previously treated unresectable metastatic anal cancer (NCI9673): a multicentre, single‐arm, phase 2 study. Lancet Oncol. 2017;18(4):446‐453. 28223062 10.1016/S1470-2045(17)30104-3 PMC5809128

[13] Ott PA , Piha‐Paul SA , Munster P , et al. Safety and antitumor activity of the anti‐PD‐1 antibody pembrolizumab in patients with recurrent carcinoma of the anal canal. Ann Oncol. 2017;28(5):1036‐1041. 28453692 10.1093/annonc/mdx029 PMC5406758

[14] Glynne‐Jones R , Nilsson PJ , Aschele C , et al. Anal cancer: ESMO‐ESSO‐ESTRO clinical practice guidelines for diagnosis, treatment and follow‐up. Radiother Oncol. 2014;111(3):330‐339. 24947004 10.1016/j.radonc.2014.04.013

[15] Petrelli F , Ghidini A , Inno A , Barni S . Mitomycin‐C+fluoropyrimidines in heavily pretreated metastatic colorectal cancer: a systematic review and evidence synthesis. Anticancer Drugs. 2016;27(6):488‐495. 27186954 10.1097/CAD.0000000000000363

[16] Sawada N , Ishikawa T , Fukase Y , et al. Induction of thymidine phosphorylase activity and enhancement of capecitabine efficacy by taxol/taxotere in human cancer xenografts. Clin Cancer Res. 1998;4(4):1013‐1019. 9563897

[17] Franchi F , Barone C , Seminara P , et al. 5‐Flugrouracil (FU) and mitomycin c (MMC) in the management of colorectal carcinoma. Part II.in vitro activity of the two drugs in short term tumor cultures. Med Oncol Tumor Pharmacother. 1991;8(2):75‐78. 1749303 10.1007/BF02988857

[18] Flam M , John M , Pajak TF , et al. Role of mitomycin in combination with fluorouracil and radiotherapy, and of salvage chemoradiation in the definitive nonsurgical treatment of epidermoid carcinoma of the anal canal: results of a phase III randomized intergroup study. J Clin Oncol. 1996;14(9):2527‐2539. 8823332 10.1200/JCO.1996.14.9.2527

[19] Ajani JA , Winter KA , Gunderson LL , et al. Fluorouracil, mitomycin, and radiotherapy vs fluorouracil, cisplatin, and radiotherapy for carcinoma of the anal canal: a randomized controlled trial. JAMA. 2008;299(16):1914‐1921. 18430910 10.1001/jama.299.16.1914

[20] Eisenhauer EA , Therasse P , Bogaerts J , et al. New response evaluation criteria in solid tumours: revised RECIST guideline (version 1.1). Eur J Cancer. 2009;45(2):228‐247. 19097774 10.1016/j.ejca.2008.10.026

[21] Peyrade F , Cupissol D , Geoffrois L , et al. Systemic treatment and medical management of metastatic squamous cell carcinoma of the head and neck: review of the literature and proposal for management changes. Oral Oncol. 2013;49(6):482‐491. 23415727 10.1016/j.oraloncology.2013.01.005

[22] Boussios S , Seraj E , Zarkavelis G , et al. Management of patients with recurrent/advanced cervical cancer beyond first line platinum regimens: where do we stand? A literature review. Crit Rev Oncol Hematol. 2016;108:164‐174. 27931835 10.1016/j.critrevonc.2016.11.006

[23] Abbas A , Nehme E , Fakih M . Single‐agent paclitaxel in advanced anal cancer after failure of cisplatin and 5‐fluorouracil chemotherapy. Anticancer Res. 2011;31(12):4637‐4640. 22199342

[24] Kim R , Byer J , Fulp WJ , Mahipal A , Dinwoodie W , Shibata D . Carboplatin and paclitaxel treatment is effective in advanced anal cancer. Oncology. 2014;87(2):125‐132. 25012155 10.1159/000361051

[25] Sclafani F , Morano F , Cunningham D , et al. Platinum‐fluoropyrimidine and paclitaxel‐based chemotherapy in the treatment of advanced anal cancer patients. Oncologist. 2017;22(4):402‐408. 28209745 10.1634/theoncologist.2016-0241 PMC5388368

[26] Rogers JE , Ohinata A , Silva NN , Mehdizadeh A , Eng C . Epidermal growth factor receptor inhibition in metastatic anal cancer. Anticancer Drugs. 2016;27(8):804‐808. 27272412 10.1097/CAD.0000000000000383

[27] Kim DW , Byer J , Kothari N , Mahipal A , Chang YD , Kim RD . EGFR inhibitors in patients with advanced squamous cell anal carcinomas: a single‐institution experience. Oncology. 2017;92(4):190‐196. 28152526 10.1159/000452766

[28] Massarelli E , William W , Johnson F , et al. Combining immune checkpoint blockade and tumor‐specific vaccine for patients with incurable human papillomavirus 16‐related cancer: a phase 2 clinical trial. JAMA Oncol. 2019;5(1):67‐73. 30267032 10.1001/jamaoncol.2018.4051 PMC6439768

[29] Cetuximab + Avelumab or Avelumab Alone for Unresectable, Locally Advanced or Metastatic Squamous Cell Anal Carcinoma (SCCAC) Progressed After at Least One Line of Systemic Treatment (CARACAS). https://clinicaltrials.gov/ct2/show/NCT03944252. Accessed May 9, 2019.

[30] Kang EJ , Choi YJ , Kim JS , et al. Mitomycin‐C, 5‐fluorouracil, and leucovorin as a salvage therapy in patients with metastatic colorectal adenocarcinoma. Asia Pac J Clin Oncol. 2010;6(4):286‐291. 21114778 10.1111/j.1743-7563.2010.01334.x

[31] Lim DH , Park YS , Park B‐B , et al. Mitomycin‐C and capecitabine as third‐line chemotherapy in patients with advanced colorectal cancer: a phase II study. Cancer Chemother Pharmacol. 2005;56(1):10‐14. 10.1007/s00280-004-0963-2 15782313

[32] Francois E , Smith D , Dahan L , et al. Uracil‐tegafur/leucovorin and mitomycin C salvage therapy in patients with advanced colorectal cancer: a phase II study. J Chemother. 2012;24(4):207‐211. 23040684 10.1179/1973947812Y.0000000021

